# Cytokine alterations in first-episode schizophrenia and bipolar disorder: relationships to brain structure and symptoms

**DOI:** 10.1186/s12974-018-1197-2

**Published:** 2018-05-26

**Authors:** Tyler A. Lesh, Milo Careaga, Destanie R. Rose, A. Kimberley McAllister, Judy Van de Water, Cameron S. Carter, Paul Ashwood

**Affiliations:** 1 Department of Psychiatry and Behavioral Sciences, University of California at Davis, Sacramento, USA; 2 Department of Medical Microbiology and Immunology, University of California at Davis, 3146 Tupper Hall, 1 Shields Avenue, Davis, CA 95616 USA; 3 MIND Institute, University of California at Davis, 2805, 50th Street, Sacramento, CA 95817 USA; 4 Center for Neuroscience, University of California at Davis, Davis, USA; 5 Division of Rheumatology, Allergy and Clinical Immunology, University of California at Davis, Davis, CA USA

**Keywords:** Schizophrenia, Bipolar disorder, Psychosis, Cytokine, Structural MRI, Immune, Cortical thickness

## Abstract

**Background:**

Over the past 30 years, evidence has been accumulating for an immunological component to schizophrenia etiology, including genetic links to the major histocompatibility complex, microglia activation, and dysregulated cytokine profiles. However, the degree of similarity in cytokine profiles for schizophrenia and bipolar disorder, as well as the relationship between cytokine levels and brain structure, is less well understood.

**Methods:**

To address this, we recruited 69 first-episode schizophrenia-spectrum patients, 16 first-episode bipolar patients with psychotic features, and 53 healthy controls, from the UC Davis EDAPT clinic. Blood plasma was collected and analyzed for all participants with a subset of participants that also underwent structural MRI on a 1.5T GE scanner.

**Results:**

Plasma levels of interleukin (IL)-1β, IL-2, IL-6, and interferon (IFN)-γ were elevated in schizophrenia patients compared to those in controls. Patients with bipolar disorder had elevated plasma IL-10 levels compared to controls, and the two patient groups did not differ significantly on any immunological measure. Percent whole-brain gray matter was inversely correlated with IFN-γ and IL-12 levels in patients with schizophrenia, with a trend relationship between IFN-γ and IL-12 and prefrontal cortical thickness. Furthermore, psychotic symptoms were positively related to IL-1β levels in individuals with schizophrenia.

**Conclusions:**

These data suggest a partially overlapping pattern of elevated blood cytokine levels in patients with first-episode schizophrenia and bipolar disorder with psychotic features. Furthermore, our findings suggest that elevated pro-inflammatory cytokines may be particularly involved in schizophrenia etiology, given evidence of cytokine-related decreases in total gray matter.

**Electronic supplementary material:**

The online version of this article (10.1186/s12974-018-1197-2) contains supplementary material, which is available to authorized users.

## Background

Schizophrenia is a complex neurodevelopmental disorder that affects approximately 1% of the population and appears to be caused by the combination of genetic risk factors and environmental insults [1, 2]. While dopaminergic hyperfunction in the limbic system and dopaminergic hypofunction in the frontal cortex are implicated in the pathophysiology of schizophrenia, many aspects of the etiology of schizophrenia remain unknown [3]. There is a growing awareness that immune dysfunction may play a role in neurodevelopmental disorders such as schizophrenia and bipolar disorder, which share some genetic and phenotypic features [4–6]. There is rapidly growing evidence for a strong link between schizophrenia and genes that regulate both immune function and brain development located in the major histocompatibility complex (MHC) region on chromosome 6 [7–9]. Immune mediators such as cytokines and chemokines can modulate neural development [10, 11] and synapse plasticity [12], which may contribute to cognitive, behavioral, and brain structure abnormalities seen in psychotic disorders.

A significant number of studies have attempted to determine whether or not there are differential cytokine profiles in individuals with schizophrenia. A quantitative review of 62 studies that included 2298 individuals with schizophrenia and 1858 controls found that plasma/sera levels were increased for interleukin (IL)-1 receptor antagonist, soluble IL-2 receptor, and IL-6 in individuals with schizophrenia [13]. In a second meta-analysis, where 40 studies were assessed, data pointed to the presence of a subset of elevated cytokines in schizophrenia, with potential state markers during acute exacerbations and first-episode psychosis that included increased inflammatory cytokines IL-1β and IL-6, and the regulatory cytokine transforming growth factor-β (TGF-β). Importantly, these cytokine levels were normalized after therapy [14]. Potential trait markers that remained elevated following antipsychotic treatment such as elevated IL-12, interferon-γ (IFN-γ), and tumor necrosis factor-α (TNF-α) were also identified [14]. Taken together, both studies clearly show that cytokine levels are altered in the plasma/sera of individuals with schizophrenia.

A similar search for a unique cytokine profile in bipolar disorder has revealed a largely overlapping immune profile with schizophrenia. A meta-analysis of 30 studies that included 1351 individuals with bipolar disorder and 1248 controls found that plasma/sera levels were elevated for IL-6, TNF-α, soluble IL-2 receptor, IL-4, IL-10, IL-1 receptor antagonist, and soluble TNF receptor-1 [15]. Notably, IL-4 and IL-10 elevations were highly significant in this meta-analysis, but are less frequently identified in patients with schizophrenia. In contrast, IFN-γ elevations are frequently noted in patients with schizophrenia, but less so in patients with bipolar disorder. Several studies have directly compared plasma cytokine levels between these two groups, generally focusing on a small number of candidate cytokines. Kunz et al. [16] examined three cytokines (IL-6, IL-10 and TNF-α) in a chronic sample and found elevated IL-6 in schizophrenia, but not bipolar disorder, while both groups showed elevated IL-10 compared to controls, and there were no significant differences between groups for TNF-α. In contrast, IL-6 was not different between groups in a sample of 125 bipolar, 186 schizophrenia, and 244 control participants [17]. Rather, soluble TNF receptor 1 and von Willebrand factor were increased in both disorders compared to controls. A recent meta-analysis of 18 schizophrenia, 16 bipolar disorder, and 12 major depressive disorder studies revealed significant elevations of IL-6, TNF-α, IL-1 receptor antagonist, and soluble IL-2 receptor in acute episodes of all groups compared to controls. Furthermore, in chronic patients, IL-6, soluble IL-2 receptor, and IL-1β were similarly elevated in schizophrenia and bipolar groups compared to controls [18]. Taken together, these data suggest that the affective and non-affective psychoses may exhibit generally similar cytokine profiles (i.e., elevations of IL-6, IL-1β, TNF-α, soluble IL-2 receptor), with some focal differences (i.e., IL-4 and IL-10 in bipolar participants).

Because of the potential biological and therapeutic importance of any overlap in cytokines in schizophrenia and bipolar disorder with psychotic features, it is imperative to analyze cytokine profiles indicated by these meta-analyses in individuals with first-onset schizophrenia and directly compare to patient groups with bipolar disorder with psychotic features and age-matched healthy controls. In addition, the relationship between changes in plasma cytokine profiles and changes in brain structure and symptomatology should be assessed to provide insight into their potential role in the pathophysiology of psychotic disorders, rather than as non-specific peripheral markers of stress responses associated with having a mental illness. Several groups have reported such links. IL-6 gene expression in blood leukocytes, for example, was associated with decreased hippocampal volume in patients with schizophrenia [19]. In addition, an aggregate measure of pro-inflammatory cytokine levels (i.e., summed z-transformed IL-2, IFN-γ, and TNF-α) was negatively associated with prefrontal cortical thickness, particularly in individuals at risk for and who eventually developed a psychotic disorder [20]. To provide a more granular view of how particular cytokines relate to brain structure and symptomatology in patients with first-episode schizophrenia and bipolar disorder with psychotic features, we focused on a narrow range of inflammatory markers implicated in previous meta-analyses [14, 15], which included pro-inflammatory cytokines IL-1β, IL-2, IL-6, IL-12, IFN-γ, and TNF-α and the anti-inflammatory cytokines IL-4 and IL-10. The first goal of this study was to investigate any differences in cytokine levels in plasma between individuals with first-episode schizophrenia, first-episode bipolar disorder with psychotic features, and healthy controls. The second goal of the study was to reveal any associations between changes in cytokine levels and brain structure. Given previous reports [14, 15], we anticipated a largely overlapping pattern of pro-inflammatory cytokine elevation in both patient groups compared to controls. Furthermore, we predicted that pro-inflammatory cytokines would be related to both global (i.e., whole-brain gray matter) and local (i.e., prefrontal cortical thickness) brain structural abnormalities. Finally, we expected that higher levels of pro-inflammatory cytokines would be associated with increasing psychotic symptomatology.

## Methods

### Demographic and clinical characteristics

Sixty-nine first-episode schizophrenia-spectrum patients (60 schizophrenia, 8 schizoaffective, and 1 schizophreniform), 16 first-episode bipolar disorder I patients with psychotic features, and 53 healthy control subjects between 13 and 32 years of age were recruited for the present study. Table 1 outlines demographic and clinical status at the time of testing. First-episode psychosis participants were outpatients within 1 year of onset of psychotic symptoms. All participants were assessed using the Structured Clinical Interview for the DSM-IV-TR (SCID)-I/P [21]. Clinical interviews were conducted by clinicians trained to high degree of reliability (kappa> 0.80; range 0.80–1.0). First-episode participants were followed longitudinally and diagnoses were confirmed 12 months after ascertainment. Clinical ratings were collected in the patient sample using the Scale for the Assessment of Negative Symptoms (SANS; [22]), Scale for the Assessment of Positive Symptoms (SAPS; [23]), Brief Psychiatric Rating Scale (BPRS; [24]), and Global Assessment of Functioning (GAF; [25]). Duration of illness was defined as the number of days between the structural MRI and first threshold psychotic symptom presentation, which was based on all available information (i.e., parent/subject report, medical records). The majority of patients with schizophrenia and bipolar disorder were taking atypical antipsychotic medication, with only 15 individuals not taking antipsychotics (10 schizophrenia, 5 bipolar). Antipsychotic dose was converted to chlorpromazine (CPZ) equivalent form. Both retrospective and current antipsychotic doses were collected to facilitate analyses of both current medication level and cumulative exposure to antipsychotics (calculated as number of days on a given dose times dose for those days). Dose history and current medication dose were obtained via self-report and verified with caregivers and notes in the medical record when possible. Exclusion criteria for all groups included Wechsler Abbreviated Scale of Intelligence (WASI) IQ score below 70, alcohol or drug dependence or abuse within 3 months before testing, positive urine toxicology screen for illicit drugs at time of testing, use of anti-inflammatory medication, presence of significant general medical condition related to immune compromise, prior head trauma worse than a Grade I concussion, or contraindication to MRI scanning. Controls were excluded for the following additional criteria: any lifetime diagnosis of an axis I or axis II disorder or any first-degree relatives with a psychotic disorder. After a complete description of the study to the subjects, written informed consent was obtained. The study was approved by the University of California, Davis Institutional Review Board, and subjects were compensated for their participation.

**Table 1:** Demographic and clinical characteristics of the sample

	Control (*n* = 53)	Schizophrenia (*n* = 69)	Bipolar (*n* = 16)
Age: mean (SD) [minimum, maximum]	19.5 (3.3) [13, 32]	19.9 (3.5) [13, 30]	21.4 (3.4) [14, 27]
Gender (% male/female)	64/36	88/12	75/25
Subject education: mean (SD)	12.7 (2.9)	12.0 (2.2)	12.7 (1.7)
Parental education: mean (SD)	14.1 (3.2)	13.9 (2.8)	14.8 (2.6)
SANS: mean (SD)	–	10.3 (3.6)	7.5 (4.9)
SAPS: mean (SD)	–	6.2 (3.7)	2.9 (3.3)
BPRS: mean (SD)	–	42.04 (8.2)	38.1 (8.0)
GAF: mean (SD)	–	41.46 (8.5)	46.50 (13.0)
WASI: mean (SD)	115.71 (10.0)	100.49 (13.2)	100.13 (11.2)
Duration of illness: mean number of days (SD)	–	319.25 (243.0)	226.00 (204.5)
Antipsychotic dose (CPZ mg): mean (SD)	–	252.1 (192.2)	271.2 (202.7)
Antipsychotic medication: (*n*)	–		
Aripiprazole Asenapine Haloperidol Olanzapine Quetiapine Risperidone Ziprasidone More than one atypical Unmedicated		14 1 1 7 3 27 1 5 10	1 0 0 2 1 5 1 1 5

*SD* standard deviation, *SANS* Scale for the Assessment of Negative Symptoms, *SAPS* Scale for the Assessment of Positive Symptoms, *BPRS* Brief Psychiatric Rating Scale, *GAF* Global Assessment of Functioning, *WASI* Wechler Abbreviated Scale of Intelligence, *CPZ* Chlorpromazine equivalent dose

Participant demographic and clinical information is presented in Table 1. Participants with schizophrenia did not differ in age from controls (t(120) = .64, *p* = 0.522) or from participants with bipolar disorder with psychotic features (t(83) = 1.60, *p* = 0.114). However, participants with bipolar disorder were significantly older than controls (t(67) = 2.04, *p* = 0.045). The sample was comprised of significantly more males in the schizophrenia group compared to the control group (Fisher’s exact test *p* = 0.002), while the bipolar sample did not differ from either controls (*p* = 0.550) or schizophrenia patients (*p* = 0.227) in terms of gender. The groups did not differ significantly on participant education levels (all pairwise *t* tests *p* > 0.20) or parental education (all pairwise *t* test *p* > 0.29). However, the control group scored significantly higher on the WASI compared with schizophrenia (t(117) = 6.90, *p* < 0.001) and bipolar disorder patients (t(65) = 5.15, p < 0.001). Schizophrenia and bipolar patients did not differ on WASI (t(80) = .10, *p* = 0.922), GAF (t(82) = 1.48, *p* = .156), duration of illness (t(70) = 1.29, *p* = 0.203), or BPRS scores (t(69) = 1.63, *p* = 0.108). Schizophrenia patients scored higher on the SANS (t(71) = 2.42, *p* = 0.018) and SAPS (t(67) = 3.04, *p* = 0.003) compared to individuals with bipolar disorder.

Twenty-six of the individuals with schizophrenia were on monotherapy antipsychotic medication, while 22 individuals were taking an antipsychotic with 1 additional medication and 11 were taking 2 or more additional medications. Other medications included anticholinergics (*n* = 5), mood stabilizers (*n* = 12), antidepressants (*n* = 18), anxiolytics (*n* = 12), stimulants (*n* = 2), and sleep aids (*n* = 2). Of the 10 individuals with schizophrenia not taking antipsychotics, 4 were taking antidepressants and 6 were taking no medication whatsoever. Four of the individuals with bipolar disorder with psychotic features were on monotherapy antipsychotic medication, while five individuals were taking an antipsychotic with one additional medication and two individuals were taking two or more additional medications. Other medications in this group included anticholinergics (*n* = 1), mood stabilizers (*n* = 5), antidepressants (*n* = 3), anxiolytics (*n* = 1), and sleep aids (*n* = 1). Of the five individuals with bipolar disorder with psychotic features not taking antipsychotics, three were taking mood stabilizers and two were taking no medications whatsoever. Fisher’s exact test revealed significantly higher mood stabilizer use in the bipolar disorder group compared to schizophrenia (*p* = 0.01), with no significant differences in use of other medications (all *p* > 0.374). There were also no group differences in frequency of antipsychotic medication use (Fisher’s exact test *p* = 0.146) or average dose (t(68) = 0.263, *p* = 0.68).

### Immunological procedures

Peripheral blood was collected from each subject in acid-citrate dextrose Vacutainers (BD Biosciences; San Jose, Ca). Blood was processed to obtain plasma by centrifugation at 2100 rpm for 10 min, and plasma was harvested, aliquoted and stored at − 80 °C until cytokine analysis. Plasma was assessed for protein levels of IL-1β, IL-2, IL-4, IL-6, IL-10, IL-12p70, IFN-γ, and TNF-α using a Multi-Plex high-sensitivity bead set (Millipore, Saint Charles, MO; Product Number HSCYMAG60SPMX13). Samples were run at one time according to the manufacturer’s protocol, with one sample tested per subject. Briefly, 25 μL of sample was incubated with antibody-coupled fluorescent beads and then washed and incubated with biotinylated detection antibodies followed by streptavidin–phycoerythrin. The beads were then analyzed using flow-based Luminex™ 100 suspension array system (Bio-Plex 200; Bio-Rad Laboratories, Inc.). Standard curves were generated by Bio-plex Manager software to determine unknown sample concentration, and reference cytokines were provided by the manufacturer in the kit. The minimum detectable amount for the cytokines were as follows: IL-1β 0.06 pg/mL, IL-2 0.26 pg/mL, IL-4 0.42 pg/mL, IL-6 0.2 pg/mL, IL-10 0.48 pg/mL, IL-12p70 0.34 pg/mL, IFN-γ 0.18 pg/mL, and TNF-α 0.07 pg/mL. Unknown sample concentrations that were below the limit of detection for a particular cytokine were excluded from all subsequent analyses.

### Structural image procedures

A subset of participants underwent brain imaging (30 schizophrenia, 29 control, and 10 bipolar). Spoiled gradient recalled (SPGR) images were collected on a 1.5T GE Signa scanner using the following parameters: TR = 9 msec, TE = 2 msec, flip angle = 15°, field of view = 22 cm, 124 1.5-mm axial slices, and 0.86-mm^2^ in-plane resolution. Images were processed with the Freesurfer software package (version 4.3, http://surfer.nmr.mgh.harvard.edu [26, 27] using the following processing stream). First, images underwent standard Freesurfer processing, including alignment to Talairach space, brain extraction, intensity normalization, segmentation, surface generation, and parcellation. After this initial automatic processing, software developed at the UC Davis Imaging Research Center was used to identify potentially problematic regions in each image. These regions were identified using information from Freesurfer output (i.e., Freesurfer’s list of “defect” locations that the software attempted to correct) as well as image intensity considerations (i.e., high intensity voxels occurring immediately adjacent to lower intensity voxels). A custom script was then used to sequentially present these regions to the viewer, who was blind to group membership, for possible manual editing. The primary purpose of this additional procedure was to offer a systematic way of ensuring that regions posing particular difficulty to Freesurfer’s automated pipeline would be viewed and corrected if necessary. This procedure was implemented in addition to a whole-brain slice-by-slice examination in which errors in brain extraction, image intensity, segmentation, and surface generation were corrected manually according to Freesurfer documentation. Surface renderings of white and gray matter surfaces were then viewed for defects and corrected manually before final processing was completed. A final visual inspection after all manual intervention was completed confirmed that each image had been appropriately corrected.

Cortical thickness measurements were obtained for each vertex and were mapped on a common spherical coordinate system. Left and right middle frontal gyrus regions of interest were extracted a priori from each subject to evaluate the relationship between cytokine levels and cortical thickness in a region repeatedly identified as impacted in psychotic disorders. Volumetric measurements were computed using Freesurfer’s segmentation procedure and extracted for whole-brain gray matter and whole-brain white matter to provide a gross metric of brain structure. These volumetric measures were adjusted for each individual subject’s intracranial volume in order to provide a percent gray and percent white matter value that takes into account individual variation in head size.

### Statistical procedures

All statistical analyses were performed in SPSS Statistics Version 22 (IBM, Armonk, NY, USA). All tests were two-tailed with alpha set at *p* < 0.05. The majority of immunological data was significantly skewed and was analyzed using nonparametric tests. Specifically, Kruskal-Wallis tests were implemented comparing all three groups, followed by pairwise Mann–Whitney *U* tests. Spearman’s Rho was implemented for correlation analyses. In order to evaluate the potential impact of gender imbalances on cytokine levels, supplementary analyses were performed comparing cytokine levels between the schizophrenia and control group. Based on procedures described by Conover and Iman [28], cytokine data were rank transformed across groups and separate ANCOVAs were performed for each cytokine including gender as a covariate. Similarly, in order to account for the influence of age and gender on the relationship between cytokine levels and brain structure, supplementary nonparametric partial correlations were performed using Spearman’s Rho [29], controlling for age and gender within each group. To account for multiple comparisons, the Benjamini–Hochberg procedure [30] was used to maintain a false discovery rate of *p* < 0.05 for the eight cytokines examined within each analysis (i.e., cytokine levels and cytokine-brain structure correlations). Correlations between cytokine and clinical measures were considered exploratory with alpha set at *p* < 0.05. Group comparisons on measures that violated sphericity assumptions were adjusted using Greenhouse–Geisser [31].

## Results

### Immunological results

**Fig. 1:**
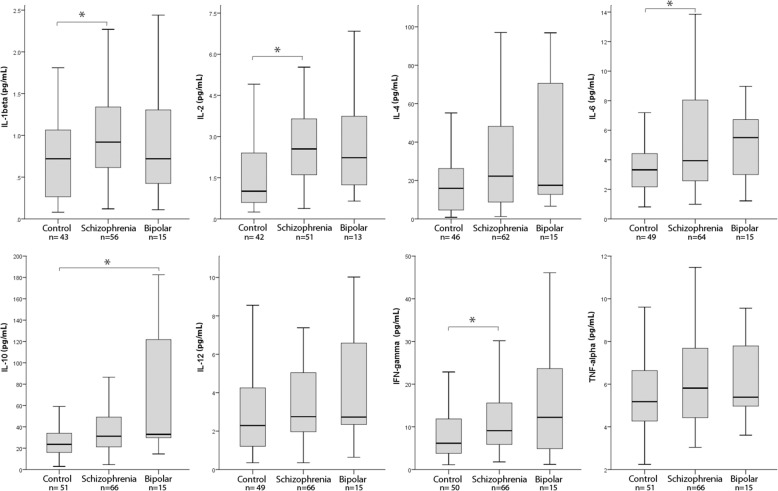
Tested cytokine levels across groups. Boxplots representing cytokine levels in each group across all eight cytokines tested. The number of subjects analyzed for each cytokine is recorded below each respective boxplot graph. The dark lines within each box represent the median, top and bottom borders of the box represent the third and first quartiles, and top and bottom whiskers represent the highest case within 1.5 times the interquartile range. Asterisks reflect *p* < .05 significance after false discovery rate correction using the Benjamini-Hochberg procedure. For ease of visualization, extreme values outside this range are excluded from these plots but are reported in Additional file 1: Table S4 in the Supplement

Examining cytokines across all three groups (see Fig. 1 for boxplots), Kruskal-Wallis tests revealed significant overall group differences for IL-2 (H(2) = 15.93, *p* < 0.001) and IL-10 (H(2) = 10.53, *p* = 0.005) after correcting for multiple comparisons. In comparison to controls, schizophrenia patients had higher levels of IL-2 (*U* = 1573.5, *p* < 0.001) and also the inflammatory cytokines IL-1β (*U* = 1565.5, *p* = 0.011), IL-6 (*U* = 1975.5, *p* = 0.018), and IFN-γ (*U* = 2076.0, *p* = 0.018) (Fig. 1). There was also a trend for increased IL-12 levels in schizophrenia but this did not reach statistical significance following correction for multiple comparisons. In addition, in comparison to controls, levels of the regulatory cytokine IL-10 were elevated in bipolar patients with psychotic features (*U* = 587, *p* = 0.002). The two patient groups did not differ significantly on any immunological measure following correction for multiple comparisons, although there was a trend for higher IL-10 levels in bipolar disorder compared to schizophrenia participants (*U* = 648, *p* = 0.063). Taken together, these results show a strong pro-inflammatory phenotype in the serum of individuals with schizophrenia compared to controls, with bipolar participants characterized by nonsignificant pro-inflammatory elevations and uniquely elevated IL-10.

Given the presence of a significant gender imbalance in the control versus schizophrenia group, supplementary analyses were conducted using rank transformed ANCOVA and including gender as a covariate. Including gender as a covariate on the ranked data had a relatively minor impact on the results: IL-1β (F_1,96_ = 6.62, *p* = 0.012), IL-2 (F_1,90_ = 15.14, *p* < 0.001), IL-6 (F_1,110_ = 5.59, *p* = 0.020), and IFN-γ (F_1,113_ = 4.17, *p* = 0.043). However, IL-6 and IFN-γ narrowly miss the cutoff threshold for Benjamini-Hochberg FDR correction.

### Relationship of immune measures with structural and clinical measures

A trend inverse correlation was identified between percent whole-brain gray matter and IL-12 (*r*_s_ = −.502, *p* = 0.009) in the control group, after correcting for multiple comparisons. Schizophrenia patients showed a significant inverse relationship between percent whole-brain gray matter and IFN-γ (*r*_s_ = −.515, *p* = 0.004). Following correction for multiple comparisons, no correlations of cytokines with cortical thickness were observed. In schizophrenia patients, there was a trend towards an inverse correlation for left middle frontal gyrus thickness and IFN-γ (*r*_s_ = −.424, *p* = 0.024) but this did not reach significance following correction for multiple comparisons. No significant correlations were identified in the bipolar participants, although there was a trend for an inverse relationship between percent whole-brain white matter and IL-1β (*r*_s_ = −.624, *p* = 0.054).

Given that some participant characteristics may influence the relationship between cytokine levels and brain structure, follow-up analyses were performed using nonparametric Spearman’s Rho partial correlations controlling for age and gender within each group. After controlling for age and gender, the control group did not show any significant relationships between brain structure and cytokine levels. The relationship between IFN-γ and percent whole-brain gray matter remained significant (*r*_s_ = −.567, *p* = 0.003) in the schizophrenia group, with the additional finding of a significant negative relationship between IL-12 and percent whole-brain gray matter (*r*_s_ = −.508, *p* = 0.008). Controlling for age and gender did not alter findings for individuals with bipolar disorder with psychotic features. Additional file 1: Table S1 presents cytokine-structure correlations without covarying for age and gender, while Additional file 1: Table S2 presents cytokine–structure correlations controlling for these variables.

In terms of clinical symptomatology, a significant positive relationship was identified between SAPS scores and IL-1β (*r*_s_ = .367, *p* = 0.012) in the schizophrenia group. In patients with bipolar disorder, a significant inverse relationship between SANS scores and IL-2 (*r*_s_ = −.660, *p* = 0.020) was identified. Notably, correlations with symptomatology (Additional file 1: Table S3) were exploratory and would not survive correction for multiple comparisons.

### Relationship of immune measures and antipsychotic medication

Given that antipsychotic medications may alter immune profiles [32], we examined the relationship of antipsychotic medication and cytokine levels. Ten of 69 individuals with schizophrenia and 5 of 16 individuals with bipolar disorder were not taking antipsychotic medication at the time of the blood draw. Mann–Whitney *U* tests between patients taking medications and those who were unmedicated at the time of blood draw revealed no effect of current antipsychotic medication on cytokine levels in the schizophrenia sample (all *p* > 0.126) but did show higher IL-2 levels in unmedicated patients with bipolar disorder compared to medicated patients with bipolar disorder (*U* = 0, *p* = 0.003). However, given the very small number of unmedicated patients with schizophrenia (*n* = 10) and bipolar disorder with psychotic features (*n* = 5), these results should be interpreted with caution. To further explore possible relationships between medication and cytokine levels, correlations were first computed between cytokine level and current antipsychotic medication dose (Additional file 1: Table S3). In patients with schizophrenia, a significant positive association was identified between antipsychotic dose and both IL-2 (*r*_s_ = .307, *p* = 0.040) and IL-10 (*r*_s_ = .273, *p* = 0.036). Additionally, cumulative exposure to antipsychotic medication over the lifespan (dose times duration) was significantly related to IL-6 (*r*_s_ = .271, *p* = 0.041) and IL-12 (*r*_s_ = .274, *p* = 0.036) in patients with schizophrenia. Given that cumulative antipsychotic exposure was highly correlated with duration of illness (*r*_s_ = .490, *p* < 0.001), one concern was that antipsychotic relationships to IL-6 and IL-12 may simply reflect alterations associated with the length of the illness. Notably, IL-6 levels were significantly associated with duration of illness (*r*_s_ = .368, *p* = 0.006), although IL-12 levels were not (*r*_s_ = .044, *p* = 0.748). This pattern of results suggests that while the relationship between cumulative antipsychotic exposure and IL-6 may be confounded by duration of illness, higher IL-12 values in individuals with greater exposure to antipsychotics may be interpreted with greater confidence as relating to medication effects. Secondary analyses using nonparametric Spearman partial correlations covarying for duration of illness agree with this interpretation given that IL-6 levels are no longer significantly related to antipsychotic exposure (*r*_s_ = .122, *p* = 0.430) while IL-12 remains significant (*r*_s_ = .290, *p* = 0.037). Neither current dose nor cumulative exposure metrics showed a relationship with cytokine levels in individuals with bipolar disorder. Finally, it should be noted that analyses of medication effects were exploratory and would not survive correction for multiple comparisons.

## Discussion

In order to start to test the hypothesis that immune activation may play a role in the etiology of schizophrenia and/or bipolar disorder or in their continued pathophysiology, we measured peripheral cytokine protein levels and correlated those changes with clinical symptoms and brain structure in individuals with schizophrenia, bipolar disorder with psychotic features, and healthy controls. Our findings are consistent with this hypothesis since cytokines are indeed elevated in the plasma of both schizophrenia and bipolar disorder compared to controls and those elevations correlate with the severity of clinical symptoms and decreases in whole-brain gray matter volume, at least in schizophrenia. Compared to controls, there were differences in these measures between bipolar disorder and schizophrenia, suggesting that the immune contribution to these disorders is overlapping but with distinct features. Together, our results are consistent with the growing body of work implicating ongoing immune dysregulation in the pathophysiology of psychotic disorders.

Cytokines mediate signals between the immune and nervous system and can help shape both neuronal responses and subsequent behaviors as well as brain growth early in development. Examining cytokines that regulate neuroimmune communication in health and disease can provide insight into the potential role of the immune response in individuals with schizophrenia.

The pro-inflammatory cytokines IL-1β, IL-6, and IFN-γ were increased in first-episode schizophrenia compared with controls, similar to previous findings described in meta-analytic studies [14]. Because these cytokines are acute phase cytokines that promote inflammation, these results indicate a pro-inflammatory state in the periphery in schizophrenia that could be the result of and/or contribute to the ongoing the disease process. We also found that IL-2 was increased in schizophrenia, again consistent with previous reports [33, 34]. Notably, soluble IL-2 receptor (sIL-2R) levels have been reported by others to be consistently increased in schizophrenia [14]. As sIL-2R can bind IL-2 with an affinity similar to that of cell membrane IL-2 receptor, it is possible that sIL-2R acts in an immunosuppressive manner to block IL-2 interacting with its receptors, suggesting that the “net” or biological effect of elevated IL-2 found in our study would be neutral. Together, these results are consistent with the possibility that pro-inflammatory cytokines contribute to the ongoing pathophysiology of schizophrenia.

While median levels of IL-6, IFN-γ, and IL-12 in the bipolar disorder group were near or above levels in the schizophrenia group, none were significantly different compared to either group. However, the anti-inflammatory cytokine IL-10 was only significantly elevated in first-episode bipolar disorder, consistent with a previous meta-analysis [15]. These elevations in IL-10 could be indicative of immunosuppression, or it could reflect a compensatory response to the elevations in the pro-inflammatory cytokines.

Changes in peripheral cytokine levels in young adults with first-episode psychosis likely reflect ongoing symptomology and brain structural changes associated with the disease process. Although the cause of these elevated cytokines is unknown, it is possible that they reflect ongoing immune dysregulation that started in gestation. Maternal infection during gestation is a risk factor for schizophrenia [35], and direct injection of inflammatory cytokines into a pregnant mouse results in structural brain abnormalities as well as behavioral and cognitive abnormalities in the offspring [1, 2]. Consistent with this idea, offspring from mouse models of maternal immune activation exhibit prominent immune changes that persist into adulthood including changes in macrophage activation, decreased regulatory T cells, and increased T helper 17 (T_H_17) cells that produce IL-17, as well as changes in levels of several pro- and anti-inflammatory cytokines within different regions of the brain [36–40]. In addition, ongoing activation of myeloid cells and production of inflammatory cytokines were directly related to behavioral outcomes in the MIA model [36]. In humans, recent studies have shown that a heightened immune response during pregnancy can modulate and influence the developing immune system leading to changes in the offspring that last into adulthood [41]. Thus, immune dysfunction noted in some individuals with schizophrenia, including altered plasma cytokine levels reported here, could be related to prenatal exposures in a manner suggested by the MIA preclinical model.

Correlations between elevations in some of these cytokines and brain structure suggest that at least some of the cytokines may contribute to the pathophysiology in schizophrenia. The strongest association was an inverse relationship between pro-inflammatory cytokines IFN-γ and IL-12 and percent whole-brain gray matter in individuals with schizophrenia. These results are consistent with data showing an inverse relationship between another pro-inflammatory cytokine, IL-6, and hippocampal gray matter volume in healthy middle-aged adults [42]. Although one cannot directly compare protein found in the plasma and mRNA cytokine expression levels in cells, it is interesting to note that IL-6 gene expression in blood leukocytes is inversely associated with hippocampal volume in patients with schizophrenia [19]. Increased IL-1β mRNA levels in leukocytes in patients with schizophrenia have also been linked to decreased Broca’s area volume and poorer verbal fluency [43]. Finally, Dieset et al. [44] evaluated seven peripheral endothelial markers in patients with bipolar disorder and schizophrenia and found the most significant relationship between von Willebrand factor and basal ganglia size. Thus, it is intriguing to evaluate whether an increase in pro-inflammatory cytokine levels in the periphery may provide a way to measure immune dysregulation in individuals that might translate to differences in the CNS. Future studies will need to determine the relationship between peripheral cytokine levels and expression levels of IL-6, IL-8, and to some extent IL-1β, which have been shown to be elevated in the DLPFC in postmortem tissue from patients with schizophrenia [45]. Other markers of inflammation, such as cellular activation of microglia, have also been found in patients with schizophrenia, particularly in the hippocampus and whole-brain gray matter [46, 47]. Whether this activation is associated with increased pro-inflammatory cytokine production needs further testing. In contrast to schizophrenia, the lack of a significant relationship between cytokine elevations and brain structure in the bipolar subjects may indicate that inflammation is not a key factor in bipolar disorder. However, there was a trend for an inverse relationship between IL-1β and whole-brain white matter, which suggests the small sample size may have limited our ability to detect these effects.

The relationship between cytokine levels measured in blood plasma and brain structure has not been adequately studied, particularly in first-episode samples. Our results suggest that pro-inflammatory cytokine elevations, particularly IFN-γ and IL-12, may be linked to whole-brain gray matter volume reductions. Given that several cytokines, including IFN-γ, may alter the permeability of the blood brain barrier [48], elevations in pro-inflammatory cytokines in the periphery could influence brain development and induce structural changes. IFN-γ activation of inflammatory T cells and antigen-presenting cells along with the regulation of MHC class 2 genes could result in a tonically active immune response, which in turn could alter neuronal repair and growth, causing decreases in gray matter volume.

In terms of symptomatology, elevated IL-1β in the schizophrenia sample was linked to increased positive symptoms on the SAPS. Because patients experience fluctuations in psychotic symptoms during the course of their disease, this finding suggests that increased IL-1β may fluctuate over time in synchrony with symptom exacerbations. While longitudinal studies are needed to truly evaluate this hypothesis, the theory is consistent with findings that IL-1β as well as IL-6 and TGF-β may reflect state markers for schizophrenia [14]. In contrast, IL-2 was negatively correlated with negative symptoms (SANS) in bipolar participants, which is more difficult to interpret. Paradoxically, Igue and colleagues [49] identified an inverse relationship whereby increased levels of sIL-2R were related to a decrease in positive symptoms for individuals with schizophrenia treated with quetiapine. The finding of increased IL-2 levels relating to lower levels of negative symptoms suggests that immune imbalances relate to a wide spectrum of symptomatology.

Changes in cytokine levels were also associated with antipsychotic medication, such that higher IL-2 and IL-10 levels were related to higher antipsychotic doses at the time of the scan in the schizophrenia sample. In addition, higher cumulative exposure to antipsychotics was associated with higher IL-6 and IL-12 levels, although IL-6 was no longer significant after controlling for duration of illness. Increased IL-10 has been linked to atypical antipsychotic treatment in mouse models exposed to lipopolysaccharide (LPS) and polyinsinic-polycytidylic acid (Poly I:C) [50], as well as in vitro studies using Poly I:C stimulated blood samples from patients with schizophrenia [51]. Stimulated macrophages and peripheral blood mononuclear cells treated with a range of antipsychotics have also shown increased release of IL-10 [52, 53]. These findings are consistent with many recent clinical studies in schizophrenia and bipolar disorder suggesting an increase in anti-inflammatory cytokines associated with antipsychotic treatment, which may play a protective or ameliorative role in treatment. For example, Sobis and colleagues [54] treated 17 chronic schizophrenia patients with aripiprazole over 1 month and identified significant reductions in a range of pro-inflammatory cytokines with a sole increase in IL-10. In contrast, a study conducted in antipsychotic-naïve first-episode schizophrenia patients identified decreased IL-10 and IL-1RA associated with 6 weeks of atypical antipsychotic treatment [55]. Furthermore, a recent meta-analysis of 23 in vivo studies (762 subjects) using pre-post designs concluded that antipsychotic treatment increases IL-2R and IL-12 levels, with decreases in IL-1β and IFN-γ [56]. While our findings of increases in IL-12 are consistent with this meta-analysis, we did not see antipsychotic-related decreases in IL-1β or IFN-γ. Ultimately, individual studies evaluating antipsychotic effects on cytokine levels are mixed, which may be due to the large variability in methodologies used, including exposure in animal models versus humans, stimulated blood samples from healthy versus psychiatric groups, specificity of antipsychotic tested, sera tested in longitudinal versus cross-sectional designs, and sample differences in terms of chronicity. While recent meta-analyses have moved the field forward, more work needs to be done to truly unravel the relationship of antipsychotic medications with cytokine levels.

Although we tried to account for potentially confounding factors in the current study, several limitations must be considered. The relatively small sample size of bipolar subjects may have limited our ability to detect the full extent of cytokine alterations in this sample, particularly with respect to schizophrenia subjects given that both groups showed cytokine elevations. While the patient groups did not differ in terms of antipsychotic exposure, the use of mood stabilizers was more frequent in the bipolar group. Since mood stabilizer medication has been linked to lower pro-inflammatory cytokine levels (i.e., IL-6) (for review see [57]), this may have further normalized cytokine levels in the bipolar group. In bipolar participants, only IL-10 levels survived correction for multiple comparisons, while other cytokines that have been consistently implicated in the disorder were not detected (i.e., IL-4 and TNF-α). In addition, the schizophrenia and control groups were imperfectly matched, with a higher percentage of males in the schizophrenia group. Supplementary analyses covarying for gender suggested a minimal impact of gender on the results, although such a small group of females may not be fully representative of that patient group. Similarly, while the bipolar group was slightly older, age and gender were included as a covariate in cytokine-structure relationships in an attempt to statistically adjust for this difference. It should also be reiterated that evaluation of relationships between cytokine levels and both antipsychotic medication and clinical symptomatology was exploratory and not corrected for multiple comparisons.

## Conclusions

In summary, these data suggest a partially overlapping pattern of elevated inflammatory cytokines in patients with first-episode schizophrenia and bipolar disorder. Furthermore, our findings suggest that higher levels of these pro-inflammatory cytokines likely play an important role in schizophrenia pathophysiology, particularly given the evidence of cytokine-related decreases in percent whole-brain gray matter. The field could clearly benefit from future studies that test the use of adjunctive anti-inflammatory treatments as well as the use of peripheral biomarkers to identify “immune-vulnerable” subgroups of individuals who may benefit the greatest from this treatment. Finally, the role of antipsychotic medications and episodic changes in cytokine levels requires further study to understand the course of inflammation in individuals with affective and non-affective psychosis.

## Additional file

Additional file 1: **Table S1.** Correlations between cytokine levels and brain structure. **Table S2.** Partial correlations between cytokine levels and brain structure controlling for age and gender. **Table S3.** Correlations between cytokine levels and clinical measures. **Table S4.** Extreme values excluded in Fig. 1 solely for graphical representation. (DOCX 25 kb)

## Abbreviations

BPRS: Brief Psychiatric Rating Scale
CPZ: Chlorpromazine
DLPFC: Dorsolateral prefrontal cortex
EDAPT: Early Detection and Preventative Treatment
GAF: Global Assessment of Functioning
IFN: Interferon
IL: Interleukin
LPS: Lipopolysaccharide
MHC: Major histocompatibility complex
MRI: Magnetic resonance imaging
Poly I:C: Polyinsinic-polycytidylic acid
SANS: Scale for the Assessment of Negative Symptoms
SAPS: Scale for the Assessment of Positive Symptoms
SCID: Structured Clinical Interview for the DSM-IV-TR
TGF: Transforming growth factor
TNF: Tumor necrosis factor
WASI: Wechsler Abbreviated Scale of Intelligence
