# Supplementary material for: Cytokine alterations in first-episode schizophrenia and bipolar disorder: relationships to brain structure and symptoms
Source: J Neuroinflammation. 2018 May 26;15:165. doi: 10.1186/s12974-018-1197-2 (PMC5970482; doi:10.1186/s12974-018-1197-2)
Supplement: Supplementary file 1 — Table S1. Correlations between cytokine levels and brain structure. Table S2. Partial correlations between cytokine levels and brain structure controlling for age and gender. Table S3. Correlations between cytokine levels and clinical measures. Table S4. Extreme values excluded in Fig. 1 solely for graphical representation. (DOCX 25 kb) [file 12974_2018_1197_MOESM1_ESM.docx]

Supplementary Materials

Table S1: Correlations Between Cytokine Levels and Brain Structure

| **Control** | | | | | | | | | |
| --- | --- | --- | --- | --- | --- | --- | --- | --- | --- |
|  | **IL-1β** | **IL-2** | **IL-4** | **IL-6** | **IL-10** | **IL-12** | **IFN-γ** | **TNF-α** |  |
| Whole-brain Percent Gray Matter | -0.054 | -0.420 | -0.188 | -0.296 | -0.255 | -0.502 | -0.361 | 0.002 |  |
| Whole-brain Percent White Matter | 0.233 | 0.000 | 0.266 | 0.153 | 0.157 | -0.049 | 0.024 | 0.315 |  |
| Left Middle Frontal Gyrus Thickness | -0.145 | 0.155 | -0.086 | -0.132 | 0.038 | -0.044 | 0.072 | -0.057 |  |
| Right Middle Frontal Gyrus Thickness | -0.151 | -0.028 | 0.044 | -0.204 | -0.030 | -0.003 | -0.087 | -0.096 |  |
| **Schizophrenia** | | | | | | | | | |
|  | **IL-1β** | **IL-2** | **IL-4** | **IL-6** | **IL-10** | **IL-12** | **IFN-γ** | **TNF-α** |  |
| Whole-brain Percent Gray Matter | -0.031 | 0.035 | -0.110 | 0.018 | 0.116 | -0.360 | -0.515^*^ | -0.257 |  |
| Whole-brain Percent White Matter | -0.103 | -0.080 | 0.068 | 0.141 | 0.167 | 0.079 | -0.140 | -0.047 |  |
| Left Middle Frontal Gyrus Thickness | -0.089 | -0.098 | -0.265 | -0.063 | -0.126 | -0.381 | -0.424 | -0.215 |  |
| Right Middle Frontal Gyrus Thickness | 0.035 | 0.120 | -0.199 | 0.150 | -0.070 | -0.270 | -0.071 | 0.036 |  |
| **Bipolar Disorder with Psychotic Features** | | | | | | | | | |
|  | **IL-1β** | **IL-2** | **IL-4** | **IL-6** | **IL-10** | **IL-12** | **IFN-γ** | **TNF-α** |  |
| Whole-brain Percent Gray Matter | 0.479 | 0.381 | -0.182 | 0.418 | -0.139 | 0.103 | 0.188 | -0.073 |  |
| Whole-brain Percent White Matter | -0.624 | -0.429 | -0.067 | -0.600 | -0.248 | -0.418 | -0.273 | -0.213 |  |
| Left Middle Frontal Gyrus Thickness | 0.406 | 0.143 | -0.267 | 0.539 | -0.152 | 0.224 | 0.067 | 0.103 |  |
| Right Middle Frontal Gyrus Thickness | 0.370 | 0.262 | -0.225 | 0.539 | -0.261 | 0.115 | 0.030 | 0.201 |  |

Table S1 Legend: Values represent Spearman rho correlations and values marked with * survive correction for the eight cytokines tested based on Benjamini-Hochberg false discovery rate.

Table S2: Partial Correlations Between Cytokine Levels and Brain Structure Controlling for Age and Gender

| **Control** | | | | | | | | |
| --- | --- | --- | --- | --- | --- | --- | --- | --- |
|  | **IL-1β** | **IL-2** | **IL-4** | **IL-6** | **IL-10** | **IL-12** | **IFN-γ** | **TNF-α** |
| Whole-brain Percent Gray Matter | 0.160 | -0.274 | -0.012 | -0.177 | -0.104 | -0.345 | -0.255 | 0.135 |
| Whole-brain Percent White Matter | 0.226 | 0.058 | 0.269 | 0.156 | 0.128 | -0.151 | 0.042 | 0.385 |
| Left Middle Frontal Gyrus Thickness | -0.067 | 0.198 | -0.006 | -0.094 | 0.158 | 0.126 | 0.108 | -0.079 |
| Right Middle Frontal Gyrus Thickness | -0.084 | 0.029 | 0.130 | -0.163 | 0.048 | 0.129 | -0.048 | -0.076 |
| **Schizophrenia** | | | | | | | | |
|  | **IL-1β** | **IL-2** | **IL-4** | **IL-6** | **IL-10** | **IL-12** | **IFN-γ** | **TNF-α** |
| Whole-brain Percent Gray Matter | -0.251 | -0.095 | -0.133 | 0.030 | 0.027 | -0.508* | -0.567* | -0.256 |
| Whole-brain Percent White Matter | 0.139 | 0.092 | 0.094 | 0.188 | 0.325 | 0.192 | -0.213 | -0.085 |
| Left Middle Frontal Gyrus Thickness | -0.255 | -0.211 | -0.291 | -0.063 | -0.217 | -0.475 | -0.436 | -0.201 |
| Right Middle Frontal Gyrus Thickness | -0.097 | 0.051 | -0.219 | 0.179 | -0.160 | -0.358 | -0.052 | 0.092 |
| **Bipolar Disorder with Psychotic Features** | | | | | | | | |
|  | **IL-1β** | **IL-2** | **IL-4** | **IL-6** | **IL-10** | **IL-12** | **IFN-γ** | **TNF-α** |
| Whole-brain Percent Gray Matter | 0.589 | 0.468 | 0.412 | 0.357 | 0.079 | 0.120 | 0.252 | -0.331 |
| Whole-brain Percent White Matter | -0.739 | -0.503 | -0.720 | -0.599 | -0.559 | -0.520 | -0.336 | -0.078 |
| Left Middle Frontal Gyrus Thickness | 0.472 | 0.152 | 0.122 | 0.560 | -0.040 | 0.183 | -0.060 | -0.086 |
| Right Middle Frontal Gyrus Thickness | 0.416 | 0.292 | 0.162 | 0.547 | -0.181 | 0.051 | -0.089 | 0.053 |

Table S2 Legend: Values represent nonparametric Spearman rho partial correlations controlling for age and gender. Values marked with * survive correction for the eight cytokines tested based on Benjamini-Hochberg false discovery rate.

Table S3: Correlations Between Cytokine Levels and Clinical Measures

| **Schizophrenia** | | | | | | | | | |
| --- | --- | --- | --- | --- | --- | --- | --- | --- | --- |
|  | **IL-1β** | **IL-2** | **IL-4** | **IL-6** | **IL-10** | **IL-12** | **IFN-γ** | **TNF-α** |  |
| SANS | -0.027 | -0.118 | 0.032 | 0.015 | 0.030 | 0.120 | 0.096 | 0.069 |  |
| SAPS | 0.367^*^ | 0.055 | -0.184 | 0.159 | -0.024 | 0.047 | 0.026 | 0.166 |  |
| BPRS | 0.109 | -0.050 | -0.138 | -0.022 | 0.016 | -0.077 | -0.116 | -0.055 |  |
| GAF | -0.021 | 0.103 | 0.218 | -0.052 | 0.094 | 0.003 | 0.072 | -0.010 |  |
| Duration of Illness | 0.141 | -0.025 | 0.102 | 0.368^*^ | 0.103 | 0.044 | -0.038 | -0.033 |  |
| Cumulative Antipsychotic Exposure | 0.105 | 0.215 | 0.198 | 0.271^*^ | 0.206 | 0.274^*^ | 0.138 | 0.166 |  |
| Antipsychotic Dose | 0.031 | 0.307^*^ | 0.232 | 0.049 | 0.273^*^ | 0.209 | -0.027 | 0.000 |  |
| **Bipolar Disorder with Psychotic Features** | | | | | | | | | |
|  | **IL-1β** | **IL-2** | **IL-4** | **IL-6** | **IL-10** | **IL-12** | **IFN-γ** | **TNF-α** |  |
| SANS | 0.034 | -.660^*^ | -0.183 | -0.075 | 0.029 | -0.192 | -0.305 | 0.011 |  |
| SAPS | 0.001 | 0.344 | -0.038 | 0.269 | -0.136 | 0.238 | 0.184 | 0.385 |  |
| BPRS | -0.471 | -0.392 | -0.189 | -0.336 | -0.050 | -0.050 | -0.413 | 0.270 |  |
| GAF | 0.303 | 0.344 | 0.200 | 0.147 | -0.221 | -0.111 | 0.295 | -0.253 |  |
| Duration of Illness | 0.385 | 0.212 | -0.070 | 0.678^*^ | 0.200 | 0.259 | 0.028 | 0.046 |  |
| Cumulative Antipsychotic Exposure | -0.363 | -0.329 | -0.189 | -0.279 | -0.235 | -0.358 | -0.323 | 0.057 |  |
| Antipsychotic Dose | -0.267 | -0.133 | -0.112 | -0.086 | -0.291 | -0.311 | -0.185 | 0.235 |  |

Table S3 Legend: SANS, Scale for the Assessment of Negative Symptoms; SAPS, Scale for the Assessment of Positive Symptoms; BPRS, Brief Psychiatric Rating Scale; GAF, Global Assessment of Functioning

Figure 1 depicts cytokine levels across subjects but excludes a small number of extreme values to maintain consistent scaling and ease visualization. Given that nonparametric tests were used throughout, these extreme values were nonetheless used in all analyses. The number of subjects excluded for graphical purposes only are as follows:

Table S4: Extreme values excluded in Figure 1 solely for graphical representation.

| Cytokine | Control | Schizophrenia | Bipolar Disorder |
| --- | --- | --- | --- |
| IL-1β | 0 | 4 | 0 |
| IL-2 | 0 | 2 | 0 |
| IL-4 | 3 | 5 | 1 |
| IL-6 | 0 | 4 | 0 |
| IL-10 | 4 | 6 | 1 |
| IL-12 | 0 | 5 | 0 |
| IFN-γ | 1 | 7 | 1 |
| TNF-α | 0 | 2 | 0 |
